# Mortality and disability-adjusted life years for smoking-attributed cancers from 1990 to 2019 in the north Africa and middle east countries: a systematic analysis for the global burden of disease study 2019

**DOI:** 10.1186/s12885-023-10563-5

**Published:** 2023-01-24

**Authors:** Leila Rezakhani, Mitra Darbandi, Zahra Khorrami, Shima Rahmati, Fatemeh Khosravi Shadmani

**Affiliations:** 1 Fertility and Infertility Research Center, Health Technology Institute, Kermanshah University of Medical Sciences, Kermanshah, Iran; 2 Department of Tissue Engineering, School of Medicine, Kermanshah University of Medical Sciences, Kermanshah, Iran; 3 Research Center for Environmental Determinants of Health (RCEDH), Health Institute, Kermanshah University of Medical Sciences, Kermanshah, Iran; 4 Ophthalmic Epidemiology Research Center, Research Institute for Ophthalmology and Vision Science, Shahid Beheshti University of Medical Sciences, Tehran, Iran; 5 Cancer Research Center, Shahrekord University of Medical Sciences, Shahrekord, Iran

**Keywords:** Cancer, Smoking, Global burden of Disease, Mortality

## Abstract

**Aim and background:**

Smoking is a modifiable risk factor for cancers. The aim of the study is to estimate the trend of mortality and DALYs of smoking-attributed cancers in the North Africa and Middle East (NAME) countries.

**Methods:**

In this study, estimates from the Global Burden of Disease 2019 (GBD-2019) study were used to report the mortality and DALYs for 16 smoking-attributed cancers. The mortality and DALYs rates from smoking-attributed cancers were evaluated by age, sex, and the 21 countries of the NAME countries from 1990 to 2019.

**Results:**

Age standardized mortality rates (ASMR) for the 29 smoking-attributed cancers in the NAME countries in 1990 and 2019 were estimated to be 24.7 (95% Uncertainty Interval: 21.5, 27.8) and 22.4 (95%UI: 19.8, 25.4) respectively, which shows a 9.2% decrease in the three decades. DALYs/100,000 for smoking-attributed cancers was, also, estimated to be 600.3 (95%UI: 521.6, 682.6) and 515.6 (95%UI: 454.9, 585.4) respectively, which indicates a 14.1% decreased in these three decades. In the last three decades, the percentage changes in DALYs/100,000 for smoking-attributed cancers in males and females were − 0.16 and − 0.03, respectively. Plus, The percentage changes in ASMR in males and females were − 12% and 8%, respectively. Furthermore, The highest ASMR and DALYs were observed in Lebanon, Turkey, and Palestine in 2019.

**Conclusion:**

The mortality rates of cancers from smoking have increased substantially among females, in most countries of the NAME region, in recent years. The burden caused by smoking can be reduced through modifying lifestyle and applying strict laws on smoking by governments and policymakers.

## Introduction

Cancer is one of the most important concerns in global health. Global demographic characteristics predict that cancer incidence and mortality will increase in the coming years, up to an extent where there will be more than 20 million new cases in 2025 [1]. International Agency for Research on Cancer (IARC) GLOBOCAN cancer statistics in 2020 reported cancers in 185 countries or territories. of the world, 38 types of the condition, about 19 million new cases, and 10 million attributed deaths. The most common cancers diagnosed worldwide are breast, lung, and, prostate; while, the most mortality is from lung, liver, and, stomach cancers [2].

Smoking is an important risk factor that increases the risk of cancers [3]. Smoking can cause cancer of the mouth and throat, esophagus, stomach, colon, rectum, liver, pancreas, larynx, tracheal, bronchus, lung, kidney, renal pelvis, urinary bladder, and cervix as well as acute myeloid leukemia [4]. Cigarettes contain carcinogens such as polycyclic aromatic hydrocarbons and nitrosamines. Since nicotine is classified as an addictive substance, smokers use it frequently, and, therefore, tumorigenesis is more common among smokers [5].

Due to the higher rates of smoking, males showed a higher proportion of cancer than did females [6]. A 2018 study in Germany found that smoking-attributed cancer accounts for 19% of all cancers, which can be considered a high rate [7]. In 2014, Mortality of 26 types of cancers was assessed in over 30-year-old United States population with consideration of various risk factors. Among these factors, smoking had the highest percentage of deaths [8]. According to epidemiological studies, half of all deaths from lung cancer patients are due to smoking [9].

Evidence shows that the number of smokers in Low and Middle-Income Countries (LMICs) has been on the rise and is likely to continue growing [10].

Given that there are several developing countries in the North Africa and Middle East (NAME) region and the cancer rate in this region is somewhat high [11], in this study, we have evaluated the trend of deaths and burden of smoking-attributed cancers by sexes and cancer types in the region.

## Methods

### Data source

The present study was conducted using the Global Burden of Disease 2019 (GBD 2019) study obtained from the Institute for Health Metrics and Evaluation (IHME) website [12]. GBD 2019 includes estimates for 369 diseases and injuries and 87 risk factors for 23 age groups and both sexes from 1990 to 2019 in 204 countries and territories [12]. The data used in the GBD-2019 study have been collected from various sources [13].

GBD classifies causes in a hierarchy of four levels. Causes at all levels are presented in the summaries. Level 1 causes are aggregates of non-communicable diseases, injuries, and a category combining infectious diseases, maternal and neonatal disorders, and nutritional deficiencies. At level 2, there are 22 disease and injury aggregate groupings such as respiratory infections and tuberculosis, cardiovascular diseases, and transport injuries. Level 3 includes specific causes such as tuberculosis, stroke, and road injuries. In some cases, these level 3 causes are the most detailed classification, while for others a more detailed category is specified at level 4. GBD also makes estimates for impairments [12].

### Study variables

This study estimated the mortality and DALYs of 29 types of smoking-attributed cancers (in 16 categories) in the NAME region. The NAME region includes 21 countries of Afghanistan, Algeria, Bahrain, Egypt, Iraq, Iran, Morocco, Oman, Palestine, Jordan, Kuwait, Lebanon, Libya, Qatar, Saudi Arabia, Sudan, Syria, Tunisia, Turkey, United Arab Emirates, and Yemen.

The studied cancers were esophageal cancer, stomach cancer, liver cancer, larynx cancer, tracheal, bronchus, and lung cancer, breast cancer, cervical cancer, prostate cancer, colon and rectum cancer, lip and oral cavity cancer, nasopharynx cancer, pancreatic cancer, kidney cancer, bladder cancer, leukemia, other leukemia. According to the International Classification of Diseases (ICD), cancers are classified by a system of diagnostic codes used widely as a reference tool in cancer registries [14].

Smokers are defined as individuals who currently use any smoked tobacco product on a daily basis. This includes both manufactured and hand-rolled cigarettes, cigars, pipes, hookah, bidis, and all other forms of smoked tobacco [15].

### Estimation of cancer burden

The burden of disease is assessed using the disability-adjusted life year (DALYs) and the age-standardized mortality rate (ASMR). DALYs is a measure that combines years of life lost due to premature mortality (YLLs) and years of healthy life lost due to disability (YLDs). One DALY represents the loss of the equivalent of one year of full health [16]. ASMR is calculated by a weighted average of the age-specific mortality rates per 100,000 individuals [16].

### Statistical analysis

The trend and rank of ASMR and DALYs rates were assessed from 1990 to 2019 by sex and cancer types in the NAME countries. Using cancer-specific population attributable fractions (PAFs), we addressed the number of smoking-attributable deaths and DALYs for each cancer from 1990 to 2019 by sex and countries of NAME. The PAFs is the proportion of cases for an outcome that can be attributed to a certain risk factor among the entire population. The mortality and DALYs rate in 5-year age groups (from 30 to 85 years and older) by sex, age pattern, and country were assessed. Data were reported as all-ages groups and age-standardized rates per 100,000 with 95% uncertainty interval (UI) in both sexes. All statistical analyses and figures were performed using R version 4.0.2 (2020-06-22).

## Results

The total number of deaths from the smoking-attributed cancers in NAME countries were calculated to be 41,059 (95%UI: 35,751 − 46,531) and 91,978 (95%UI: 81,237 − 10,4314) in 1990 and 2019 respectively, indicating a 124% increase over the 30 years. Inversely, the attributed mortality rate from smoking-attributed cancers decreased by 9.1% in this period. The number of DALYs from smoking-attributed cancers displayed an increase from 1,100,400 (95%UI: 959,002 − 1,257,193) years in 1990 to 2,336,468 (95%UI: 2,053,920- 2,660,606) in 2019, which represents a 112% rise. In addition, it was observed that the DALYs rate decreased by 14.1%.

ASMR for smoking-attributed cancers and the related percentage changes from 1990 to 2019 have been shown in Table 1. In 1990, Bahrain ranked first in smoking-attributed cancers in both sexes, Turkey and Lebanon ranked second and third, respectively. In 2019, the highest rate of cancers caused by smoking were observed in Lebanon, Turkey, and Palestine; While Bahrain ranked ninth among the countries in the region with a 56% drop. ASMR for smoking-attributed cancers among males in NAME countries decreased from 45.7 (95%UI: 39.7–51.8) in 1990 to 40.4 (95%UI: 35.6–45.9) in 2019, which represents a decrease of 12%. Also, the ASMR among females were 3.8 (95% UI: 3.2–4.5) and 4.1 (95% UI: 3.5–4.7) in 1990 and 2019, respectively to show an increase of 8% in the last three decades. Males in Turkey had the highest mortality rate from smoking-attributed cancers with 96.8 (95% UI: 80.3-114.9) in 1990, which changed to be 72.8 (95% UI: 58.2–89.8) in 2019, indicating a reduction of 25%.

**Table 1:** Age standardized cancer attributed mortality rates of smoking and their percentage changes from 1990 to 2019

Country	Rank*		Sex	ASMR per 100 000 population (95% UI)				Percent change
	1990	2019		1990	2000	2010	2019	1990–2019
**Bahrain**	1	9	Male	91.5 (78.0- 105.3)	67.4 (57.9–77.3)	50.7 (43.4–58.3)	40.3 (31.8–50.0)	-0.56
			Female	13.3 (10.4–16.5)	11.2 (8.8–13.9)	8 (6.4–11.2)	6.5 (4.9–8.7)	-0.51
**Turkey**	2	2	Male	96.8 (80.3- 114.9)	63.2 (55.3–72.3)	81.4 (71.3–92.1)	72.8 (58.2–89.8)	-0.25
			Female	8.7 (6.7–11)	6.9 (5.6–8.4)	7.9 (6.6–9.2)	7.9 (6.1–9.9)	-0.09
**Lebanon**	3	1	Male	60.3 (48.2–74.0)	54.7 (46.6–65.6)	68.4 (56.8–90.6)	66.9 (54.9–87.4)	0.11
			Female	14.4 (11.5–17.7)	15.6 (12.7–19.1)	20.8 (17.3–27.1)	23.8 (19–32)	0.65
**United Arab Emirates**	4	4	Male	44.1 (30.6–55.8)	50.4 (38.8–62.2)	41.6 (33.7–51.2)	36.2 (28.5–44.9)	-0.18
			Female	6.9 (4.5–10.1)	8.2 (6- 10.8)	10.7 (7.4–14.8)	6.1 (4.3–8.4)	-0.12
**Palestine**	5	3	Male	59.3 (45.9–74.7)	53.5 (46.5–60.9)	54.2 (48.5–60.0)	56.6 (48.4–65.3)	-0.05
			Female	3.2 (2.2–4.6)	3.3 (2.5–4.3)	3.4 (2.7–4.2)	3.5 (2.6–4.5)	0.09
**Libya**	6	8	Male	52.4 (40.5–64.5)	50.7 (42.4–57.9)	47.9 (40.1–52.9)	47.9 (36.7–59.9)	-0.09
			Female	0.9 (0.6–1.2)	0.8 (0.6–1.1)	0.8 (0.6–1.1)	0.8 (0.5–1.1)	-0.11
**Tunisia**	7	7	Male	52.2 (43.5–61.8)	57.3 (46.9–70.2)	53.3 (38.8–70.2)	51.4 (37.6–70.3)	-0.02
			Female	1.8 (1.3–2.4)	1.9 (1.4–2.5)	1.8 (1.3–2.5)	1.7 (1.2–2.4)	-0.06
**Qatar**	8	5	Male	45.9 (36.1–56.7)	41.6 (32.9–51.5)	38.9 (31.1–47.6)	34.1 (26.0–43.0)	-0.26
			Female	2.3 (1.5–3.3)	2.4 (1.7–3.4)	2.9 (2- 4.2)	2.4 (1.7–3.4)	0.04
**Iraq**	9	6	Male	47.5 (37.8–58.8)	42.4 (32.4–54.4)	43.7 (34.1–54.2)	49.0 (38.5–57.9)	0.03
			Female	4.5 (3.2- 6)	4.5 (3.3–6.1)	5 (3.8–6.6)	5.6 (4.2–7.2)	0.24
**Jordan**	10	10	Male	41.7 (34.4–50.3)	36.5 (29.6–44.3)	41.0 (35.5–47.1)	37.2 (29.0- 46.1)	-0.11
			Female	6.5 (4.8–8.4)	6.3 (4.8–8.1)	5.9 (4.9–7.1)	6.2 (4.9–7.9)	-0.05
**Yemen**	11	11	Male	37.4 (27.2–50.9)	35.1 (25.5–47.0)	35.2 (26.7–46.1)	35.1 (26.2–47.0)	-0.06
			Female	5.1 (3.5–7.5)	5 (3.4–7.1)	5.5 (4.1–7.4)	6 (4.4- 8)	0.18
**Algeria**	12	19	Male	35.4 (28.6–43.6)	28.8 (22.9–35.4)	27.7 (22.6–33.0)	25.7 (20.4–32.3)	-0.27
			Female	2.1 (1.5- 3)	2 (1.4–2.7)	1.8 (1.3–2.3)	1.6 (1.2–2.2)	-0.24
**Iran**	13	15	Male	34.5 (29.0- 39.6)	27.5 (24.9–30.2)	27.2 (24.9–29.4)	29.1 (26.5–32.2)	-0.16
			Female	3.3 (2.5–4.2)	2.8 (2.2–3.5)	2.7 (2.2–3.3)	3.2 (2.6–3.8)	-0.03
**Syrian Arab Republic**	14	13	Male	32.1 (24.1–41.0)	32.3 (26.1–39.7)	30.3 (23.9–37.1)	28.4 (21.2–37.2)	-0.12
			Female	4.1 (2.7–5.9)	4.5 (3.3–6.1)	3.9 (2.9–5.1)	3.5 (2.5–4.8)	-0.15
**Morocco**	15	14	Male	34.2 (25.4–41.7)	33.0 (24.8–40.5)	30.3 (21.9–39.2)	32.8 (23.8–40.8)	-0.04
			Female	1.1 (0.7–1.5)	1.1 (0.7–1.4)	1 (0.7–1.4)	1 (0.7–1.3)	-0.09
**Kuwait**	16	18	Male	26.4 (23.1–29.8)	26.8 (24.2–29.3)	22.1 (19.8–24.4)	22.8 (18.1–28.0)	-0.14
			Female	4.2 (3- 5.6)	3.6 (2.7–4.5)	3.3 (2.5–4.1)	2.3 (1.6–3.1)	-0.45
**Sudan**	17	17	Male	25.9 (16.6–36.6)	24.8 (16.4–35.0)	25.4 (16.7–35.0)	25.7 (17.2–35.1)	-0.01
			Female	1.8 (1.1–2.6)	1.8 (1.1–2.6)	1.9 (1.2–2.8)	1.9 (1.2–2.6)	0.06
**Egypt**	18	12	Male	27.4 (23.5–31.1)	29.2 (25.1–33.3)	34.0 (28.8–40.1)	33.6 (24.4–45.4)	0.23
			Female	0.6 (0.5–0.8)	0.8 (0.6- 1)	1 (0.7–1.4)	1 (0.7–1.5)	0.67
**Oman**	19	21	Male	23.9 (17.9–30.0)	22.2 (18.4–26.1)	20.5 (18.2–22.9)	16.9 (14.1–20.3)	-0.29
			Female	2.1 (1.3- 3)	2.1 (1.5–2.8)	2 (1.5–2.6)	1.7 (1.3–2.3)	-0.19
**Afghanistan**	20	16	Male	21.6 (14.7–31.3)	23.1 (15.8–35.0)	26.6 (18.6–38.0)	28.5 (20.7–39.0)	0.32
			Female	1.7 (1.1–2.4)	2 (1.3–2.9)	2.4 (1.7–3.5)	2.8 (1.9 -4)	0.65
**Saudi Arabia**	21	20	Male	16.1 (12.2–20.5)	18.0 (15.7–20.5)	17.3 (15.1–20.3)	16.0 (12.9–19.7)	-0.01
			Female	1.1 (0.7–1.7)	1.6 (1.2–2.2)	1.7 (1.2–2.2)	1.7 (1.2–2.3)	0.55
**North Africa and Middle East**	-	-	Male	45.7 (39.7–51.8)	37.8 (34.5–41.2)	42.1 (38.5–45.6)	40.4 (35.6–45.9)	-0.12
			Female	3.8 (3.2–4.5)	3.5 (3–4)	3.9 (3.5–4.4)	4.1 (3.5–4.7)	0.08

* Ranks were assigned based on both sex

Table 2 shows the DALYs rate for smoking-attributed cancers. In 1990, the highest rate of DALYs was seen in the countries of Turkey, Bahrain, and Lebanon, respectively; while, in 2019 Lebanon, Turkey and Palestine had the highest DALYs rate. Females in Lebanon had the highest smoking-attributed cancer ASMR of 14.4 (95% UI: 11.5–17.7) in 1990, which with 65% increased in 2019, 23.8 (95% UI: 19.0–32) was observed. Saudi Arabia ranked 21st and 20th among countries in the region in 1990 and 2019 respectively with regard to smoking-attributed cancers.

**Table 2:** Age standardized cancer attributed DALYs rate of smoking and their percentage changes from 1990 to 2019

Country	Rank*		Sex	DALYs per 100 000 population (95% UI)				Percent change
	1990	2019		1990	2000	2010	2019	1990–2000
**Turkey**	1	2	Male	2403.1 (1970.5- 2888.5)	1552.0 (1357.8- 1783.6)	1943.5 (1684.7- 2213.6)	1713.7 (1348.0- 2141.3)	-0.29
			Female	241.9 (186.1- 305.7)	187.2 (152.2- 226.1)	202.4 (186.8- 236.9)	200.8 (23.1- 253.5)	-0.17
**Bahrain**	2	12	Male	1713.7 (1463.5- 1982.5)	1255.7 (1074.3- 1447.7)	876.4 (745.1- 1012.8)	683.2 (528.9- 861.7)	-0.60
			Female	278.4 (213.9- 352.3)	230.2 (179.0- 287.0)	147.2 (213.2- 184.2)	119.2 (23.9- 161.4)	-0.57
**Lebanon**	3	1	Male	1351.5 (1058.8- 1682.6)	1187.9 (1006.7- 1425.5)	1480.3 (1234.2- 1913.7)	1462.0 (1174.1- 1889.2)	0.08
			Female	341.3 (266.7- 423.8)	360.0 (293.8- 440.3)	470.3 (266.9- 599.4)	541.4 (23.7- 704.3)	0.59
**Libya**	4	5	Male	1211.4 (932.7- 1522.8)	1177.9 (986.4- 1344.6)	1095.8 (916.6–1209.0)	1106.2 (833.4- 1401.9)	-0.09
			Female	20.8 (13.9–29.8)	19.3 (13.3–26.7)	18.4 (13.0- 25.5)	17.9 (23.9–26.5)	0.14
**Palestine**	5	3	Male	1294.3 (988.6- 1657.5)	1142.7 (992.5- 1312.7)	1137.1 (1017.5- 1263.9)	1184.3 (1005.3–1379.0)	-0.08
			Female	69.3 (45.8- 102.2)	68.7 (51.1–91.7)	70.4 (45.3–87.8)	72.5 (23.8–93.8)	0.05
**United Arab Emirate**	6	7	Male	915.3 (645.9- 1166.7)	1026.0 (780.6- 1269.6)	850.8 (687.7- 1056.3)	746.4 (572.9- 953.8)	-0.18
			Female	158.4 (102.5- 230.9)	181.0 (131.5- 239.8)	225.3 (102.4- 308.3)	138.8 (23.5–193.0)	-0.12
**Tunisia**	7	6	Male	1146.2 (942.4- 1364.7)	1266.6 (1023.8- 1562.8)	1179.5 (842.9- 1550.3)	1133.4 (814.2- 1569.8)	-0.01
			Female	40.9 (29.1–55.1)	43.3 (31.5–58.5)	40.6 (29.9–57.3)	37.5 (23.1–54.0)	-0.08
**Iraq**	8	4	Male	1090.3 (863.9- 1357.1)	978.5 (737.1- 1282.2)	973.3 (748.0- 1234.2)	1046.2 (794.6- 1259.2)	-0.04
			Female	110.4 (80.5- 147.4)	110.9 (80.4- 151.8)	119.1 (80.8- 157.5)	128.8 (23.5- 169.2)	0.17
**Qatar**	9	11	Male	906.7 (702.1- 1129.9)	826.1 (648.7- 1032.5)	738.9 (587.3- 919.9)	597.4 (443.5- 779.7)	-0.34
			Female	52.9 (35.1–77.3)	54.1 (37.3–77.1)	58.1 (35.9–82.0)	45.9 (23.1–66.3)	-0.13
**Jordan**	10	8	Male	943.3 (777.7- 1143.3)	832.7 (668.5- 1012.4)	877.1 (762.7- 1006.4)	802.0 (620.0- 995.5)	-0.15
			Female	153.3 (113.3- 198.3)	144.8 (110.4- 189.4)	128.2 (113.4- 154.4)	131.5 (23.3- 168.6)	-0.14
**Yemen**	11	9	Male	867.7 (614.9- 1198.5)	806.4 (579.4- 1106.1)	796.2 (599.4- 1069.8)	795.0 (584.4- 1080.2)	-0.08
			Female	133.1 (89.0- 193.3)	128.4 (86.4- 183.4)	139.4 (89.2- 191.4)	149.6 (23.0- 204.4)	0.12
**Syrian Arab Republic**	12	14	Male	756.4 (565.5- 975.4)	749.6 (603.3- 933.1)	688.1 (543.8- 849.2)	648.3 (478.0- 867.5)	-0.14
			Female	101.4 (65.9- 151.9)	105.5 (74.2- 145.9)	84.8 (65.0- 113.6)	77.7 (23.9- 111.1)	-0.23
**Iran**	13	15	Male	775.4 (659.8- 886.7)	613.2 (559.9- 672.5)	599.9 (556.9- 643.7)	638.2 (584.2- 702.6)	-0.18
			Female	80.6 (61.0- 104.1)	69.0 (53.2–86.6)	65.2 (61.3–78.9)	75.0 (23.0- 88.5)	-0.07
**Morocco**	14	13	Male	858.4 (643.4- 1042.3)	826.2 (624.8- 1014.8)	761.1 (550.5- 1003.7)	790.4 (572.6- 999.1)	-0.08
			Female	29.1 (19.0- 42.5)	28.2 (19.3–40.3)	27.0 (19.2–38.8)	24.4 (23.0- 34.7)	-0.16
**Algeria**	15	18	Male	767.8 (611.4- 957.9)	621.3 (489.1- 772.7)	591.9 (477.1- 713.3)	544.8 (423.8- 686.7)	-0.29
			Female	47.0 (32.6–67.1)	42.1 (29.9–58.6)	37.0 (32.7–49.1)	32.2 (23.6–43.6)	-0.31
**Kuwait**	16	19	Male	575.6 (512.7- 644.9)	561.5 (514.5- 608.3)	445.9 (406.4- 487.2)	445.9 (344.8- 552.1)	-0.23
			Female	90.7 (64.7- 123.7)	79.0 (59.7- 100.4)	66.3 (64.2–83.3)	45.4 (23.7–60.0)	-0.50
**Egypt**	17	10	Male	691.4 (588.6- 784.3)	734.6 (628.3- 841.9)	858.3 (722.2- 1026.9)	848.9 (608.5- 1161.3)	0.23
			Female	13.5 (10.2–17.6)	16.2 (12.1–21.6)	20.6 (10.9–27.7)	20.7 (23.2–30.6)	0.53
**Sudan**	18	17	Male	597.8 (377.8–865.0)	568.9 (374.3- 818.4)	577.0 (374.0- 797.9)	571.1 (370.9- 800.3)	-0.04
			Female	45.1 (28.8–68.0)	44.5 (27.2–67.6)	47.1 (28.7–69.7)	45.0 (23.8–64.1)	0.00
**Oman**	19	21	Male	529.9 (393.6- 673.8)	487.1 (399.7- 582.4)	438.6 (391.7- 487.4)	327.6 (267.0- 407.8)	-0.38
			Female	45.9 (29.3–67.6)	45.8 (32.8–61.0)	42.1 (29.9–55.0)	35.0 (23.3–45.8)	-0.24
**Afghanistan**	20	16	Male	493.7 (320.4- 734.8)	538.4 (352.2- 828.6)	618.4 (417.2- 911.8)	659.5 (460.0- 930.1)	0.34
			Female	45.3 (27.3–68.4)	55.6 (33.9–86.3)	67.3 (27.7- 100.6)	76.9 (23.3- 113.9)	0.70
**Saudi Arabia**	21	20	Male	367.8 (273.8- 475.4)	402.4 (347.3- 464.5)	391.4 (342.2- 468.1)	369.5 (288.5- 455.3)	0.00
			Female	27.9 (18.0- 41.5)	37.8 (27.5–51.1)	38.8 (18.1–51.3)	38.0 (23.0- 53.7)	0.36
**North Africa and Middle East**	-	-	Male	1089.9 (950.2- 1243.3)	899.2(814.5- 974.2)	977.0 (893.8- 1058.4)	920.4 (810.5- 1049.6)	-0.16
			Female	97.9 (81.0- 117.0)	86.7 (74.9- 100.3)	92.6 (81.5- 104.9)	94.5 (80.9- 111.4)	-0.03

* Ranks were assigned based on both sex

The DALYs rates from smoking-attributed cancers among males in the NAME countries have decreased by 16% over three decades, from 1,089.9 (95%UI: 950.2-1,243.3) in 1990 to 920.4 (95%UI: 1,049.6-810.5) in 2019. Similarly, the DALYs rate among females has decreased by 3% over three decades, from 97.9 (95% UI: 117.0–81.0) in 1990 to 94.5 (95% UI: 111.4–80.9) in 2019. Among the countries of NAME, the highest percentage of increase in DALYs was observed among Afghan females with 70%; while, Bahraini males recorded the highest reduction (60%) in the total number of DALYs over the three decades.

**Fig. 1:**
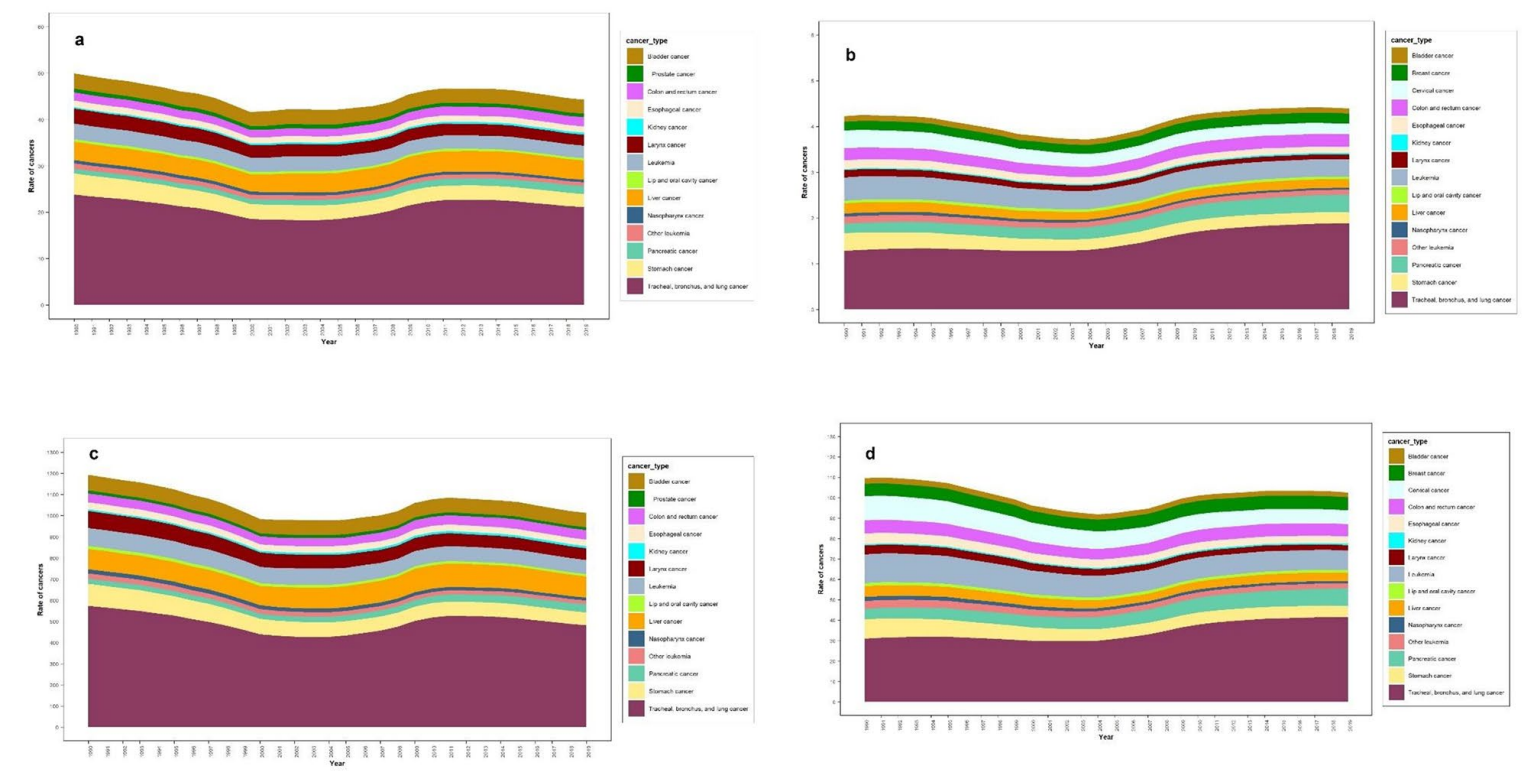
Trend of ASMR and DALYs rate of smoking-attributed cancers by cancer type, **(a)** Attributable ASMR in males, **(b)** Attributable ASMR in females, **(c)** Attributable DALYs rate in males, **(d)** Attributable DALYs rate in females

Also, tracheal, bronchus, and lung cancer claimed the highest rate of attributed smoking deaths associated with cancer in both sexes in 1990 to 2019. It accounts for 48% of all cancers related death from tracheal, bronchus, and lung cancer in males, with a steady trend from 1990 to 2019. In 1990, stomach and liver cancers had the highest smoking-attributed cancer deaths with respectively 9 and 8 percents (Fig. 1. a). The downward death trend observed in stomach cancer continued until 2019 when it reached 6%. On the other hand, there has been an increase in the death rate from liver cancer, with a 9% rate in 2019.

Similar to males, tracheal, bronchus, and lung cancer claims the highest smoking attributed cancers deaths among females. Some 30% of smoking attributed deaths among females in 1990 were associated with tracheal, bronchus, and lung cancer, and it had a 43% increase in 2019. In 1990, leukemia claimed 12% and each of the stomach and cervical cancers accounted for 9% of smoking-attributed cancer deaths. In 2019, aside from tracheal, bronchus, and lung cancer, each of pancreatitis and leukemia accounted for 9% of the smoking-attributed cancer deaths among females (Fig. 1, b). Also, The DALYs rate had a similar pattern in men(Fig. 1, c) and women(Fig. 1, d).

**Fig. 2:**
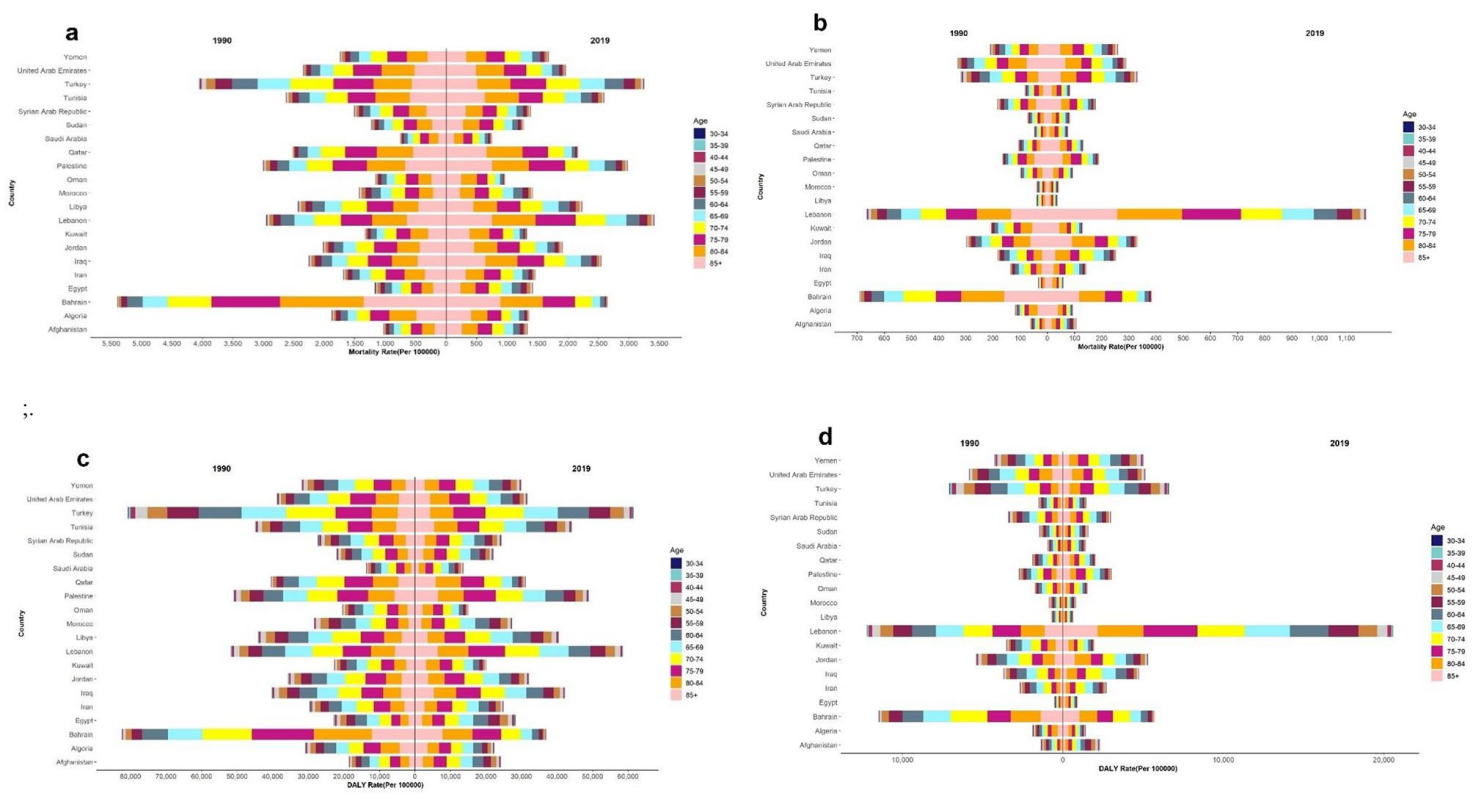
ASMR and burden of smoking-attributed cancers in age groups, **(a)** attributable ASMR in males, **(b)** attributable ASMR in females , **c)** attributable DALYs rate in males, **d)** attributable DALYs rate in females

The evaluations of age pattern of mortality and smoking-attributed cancer DALYs are shown in Fig. 2. As indicated, the ASMR among males aged 60 and over has increased over the last three decades. In addition, the highest death rate is observed in the age groups of 80 to 84 years and 85 years and above. On the other hand, the ASMR in females aged 55 and older has increased sharply. The ASMR in females has displayed a rise with increasing age and the age groups of 85 years and above as well as 80 to 84 years old had the highest mortality rate.

Bahrain and Lebanon had the highest male death rates in both 1990 and 2019. The age pattern of DALYs is similar to that of the mortality rate. Bahraini and Turkish men, as well as Lebanese and Bahraini women, had the highest DALYs rates among countries in the North Africa and Middle East region in 1990. In addition, men from Turkey and Lebanon and women from Lebanon and Turkey had the highest DALYs rates in 2019, respectively (Fig. 2).

## Discussion

In this study, using the latest GBD 2019 data, we addressed the burden of all cancers from 1990 to 2019 in the NAME countries. This study displayed a slight decrease in the DALYs/100,000 from all cancers among both sexes over the last three decades. In the NAME countries in 2019, ASMRs for smoking-attributed cancers among males and females were 40.4 and 4.1, respectively, with an increase among the female during the past three decades. The death rate per 100,000 people from 1990 to 2019 decreased by 12% in males and increased by 8% in females. Also, the declining trends in DALYs/100,000 rates were estimated at 16% and 3% during 30 years in males and females, respectively. Our results with regard to the increasing mortality trend of smoking-attributed cancers were in line with those of developed countries [17, 18].

In addition, an increasing DALYs trend is observed in some countries such as Lebanon, Egypt, Afghanistan, and Saudi Arabia. The highest ASMR and DALYs were observed in Lebanon, Turkey, and Palestine in 2019. The highest rates of ASMR and DALYs were related to the tracheal, bronchus, and lung cancer.

Previous studies have shown the high risk of smoking associated with the occurrence of cancers; a study in Iran reported that 53.5% of all lung cancer deaths were attributed to smoking [19]. Furthermore, the number of smoking-attributed cancer deaths was estimated to be 342,854 among males and 40,313 among females in China in 2014, of which, respectively, 1.8% and 50% included second-hand smoking [20]. A systematic review study in 2015 has shown that smoking is associated with pancreatic, mouth, esophagus, lung, and larynx cancers; the related mortality risk for cancers was reported to be 0.48 to 1.60 among current smokers and 0.70 to 1.68 among current cigar and/or pipe in the studies included in the systematic review [4]. A comprehensive review using the GBD-2019 study from 1990 to 2016 reported smoking as an important risk factor for all cancers in India [21]. The prevalence of smoking has been decreasing worldwide between 1990 and 2015 [12]. However, the burden of smoking was among the top five risk factors in 109 countries and regions in 2015, an increase from 88 geographic regions in 1990. Therefore, the undeniable role of smoking in cancer mortality calls for the attention of health policymakers in the region [15].

The highest mortality rates in both sexes were observed in the age groups of 80 to 84 years and ≥ 85 years. However, ASMRs in males aged 60 < and females over 55 years have increased over the last three decades. DALYs in Lebanon in all age groups have increased significantly in 2019 compared to 1990.

In the present study, the highest rates of ASMR and DALYs were related to tracheal, bronchus, and lung cancer. As studies have shown, smoking is the single most significant and preventable risk factor of lung cancer [22, 23].

Additionally, our findings demonstrate that, compared to females of the other nations in the region, the DALYs rate of smoking-attributed cancers among Lebanese females in 1990 and 2019 at different ages were reported to be much higher, with Turkey ranking next. In Oman, Bahrain, and Algeria, DALYs rates in different age groups in 2019 have decreased compared to 1990. These results show that the DALYs rate of smoking-attributed cancers has a different pattern in NAME countries. Mons et al. (2018) estimated the burden of smoking-attributed cancers in 85,072 cases (58,760 male, 26,312 female) and found that it is responsible for 19% of all incident cancers. Also, the highest population-attributable fractions (PAFs) were seen in lung cancer, with 89% of male and 83% of female lung cancer cases were attributable to smoking [7]. The standardized PAFs of cancers deaths attributable to smoking reported 22.2% and 4% of Chinese males and females, respectively [20]. In the United States, 2.6 million DALYs were lost to smoking-attributed cancer (27% of all DALYs lost to cancer) in 2011. Additionally, smoking-attributed DALYs rates were higher in males than in females (968 vs. 557 per 100,000) [24]. Other studies have also reported smoking-attributed cancer DALYs to be higher in males than in females [7, 20, 25]. Therefore, the DALYs rate of smoking-attributed cancers is significant and to reduce this burden, regional strategies are required. According to the present study, smoking-attributed cancer DALYs and ASMR have increased in females over the past three decades, indicating an increase in smoking among females. Large variations in the smoking-attributed cancer burden by sex and country reflect differences in the current and past prevalence of smoking. Smoking has been more prevalent in males than females in the past [24]. Additionally, the smoking-attributed cancer burden has been reported to differ significantly between racial and ethnic groups in previous studies [24, 26, 27]. Considering the age-sex pattern, and different mortality trends in the countries of the NAME countries, it is necessary to provide appropriate age and sex groups tailored solutions for the countries.

The present study’s findings showed that smoking-associated lung, trachea, and bronchial cancers had the highest ASMR and DALYs rates in both sexes; stomach and pancreatic cancers were also ranked next. A study by Huang in 2022 showed that smoking is a significant risk factor for the development of kidney cancer in the female population [28]. Findings from the study by Gram et al. show that compared to never-active and never-passive smokers, regular (former and current) smokers have a 21% higher overall risk of breast cancer [29]. Nevertheless, in the general population, one out of every nine breast cancers case and in the smoking population, one out of every six can be prevented by not actively smoking [29]. A large prospective cohort of about 19,000 population in 21 states of Columbia, and Puerto Rico has shown that smoking is an important prognostic factor for prostate cancer, and that the prostate cancer may be one of the leading causes of smoking-related deaths [30]. According to a meta-analysis study in 2019, compared to never-smokers, current smokers and former smokers are respectively 56% (Hazard ratio: 1.5695% CI, 1.34–1.83) and 15% (Hazard ratio: 1.15; 95% CI, 1.06–1.26) more at risk for pancreatic cancer [31]. A systematic review study has also shown that smoking is associated with all-cause mortality, and pancreatic cancers, especially of the mouth, esophagus, lungs, and larynx. [4].

According to the previous evidence, smoking and exposure to smoke are of the important causes of cancer DALYs and mortality [32]. Fortunately, smoking is a very avoidable risk factor. So far, many efforts have been made nationally and globally to combat tobacco consumption [33]. However, due to the diversity of tobacco products and the prevalence of consumption at a young age, there is still a long way to go to combat tobacco use. In addition, encouraging smoking cessation at an early age is an important way to reduce the mortality and DALYs rate as age differences in cancer incidence may be due to differences in smoking cessation rates between young, middle-aged, and elderly populations [34].

This study had some limitations. Under-reporting smoking due to social desirability bias (especially among females and the youths) may have led to underestimating the smoking-associated burden of cancers. Analyses and comparison of the smoking-attributed cancer DALYs and ASMR over 3 decades were of the present study’s strengths, which helps clarify the status of this risk factor in the NAME countries.

## Conclusion

Results of the current study indicate that smoking plays an important role in mortality and DALYs rate for a variety of cancers in NAME countries. Thus, efforts, as part of a comprehensive cancer control plan, should be reinforced to reduce, quit, and prevent of uptake of tobacco smoking, both on the individual and the population level. Further investigations should be done in specific groups and high-risk regions.

## Data Availability

The datasets generated and/or analyzed during the current study are available in the GBD study 2019 online repository, https://vizhub.healthdata.org/gbd-compare/.
